# Violation of Leggett–Garg Inequality in Perceiving Cup-like Objects and Cognitive Contextuality

**DOI:** 10.3390/e26110950

**Published:** 2024-11-05

**Authors:** Likan Zhan, Andrei Khrennikov, Yingce Zhu

**Affiliations:** 1 School of Communication Science, Beijing Language and Culture University, Beijing 100083, China; zhanlikan@hotmail.com (L.Z.); qr0709cj@163.com (Y.Z.); 2 International Center for Mathematical Modeling in Physics and Cognitive Sciences, Linnaeus University, 35195 Växjö, Sweden

**Keywords:** quantum-like modeling, Leggett–Garg inequality, classical vs. quantum probability, contextuality, hysteresis, processing uncertainties

## Abstract

This paper is devoted to an experimental investigation of cognitive contextuality inspired by quantum contextuality research. This contextuality is related to, but not identical to context-sensitivity which is well-studied in cognitive psychology and decision making. This paper is a part of quantum-like modeling, i.e., exploring the methodology of quantum theory outside of physics. We examined the bistable perception of cup-like objects, which strongly depends on experimental contexts. Our experimental data confirmed the existence of cognitive hysteresis, the important role of memory, and the non-commutative structure of cognitive observables. In physics, quantum contextuality is assessed using Bell-CHSH inequalities, and their violation is incorrectly believed to imply the nonlocality of Nature. The violation of Bell-type inequalities in cognitive and social science strongly indicates that the metaphysical implications of these inequalities are quite limited. In our experiments, modified Leggett–Garg inequalities were also significantly violated, but this only means that experimental data from experiments performed in different contexts cannot be modeled by a unique set of noncontextual, jointly distributed random variables. In our experiments, we know the empirical probability distributions measured in different contexts; thus, we can obtain much more detailed and reliable information about contextuality in human cognition by performing nonparametric compatibility tests.

## 1. Introduction

In cognitive science, psychology, decision making, and social science, the notion of context-sensitivity, including framing, is well studied [1,2,3,4,5]. This notion is similar to, but not identical to, the quantum-inspired notion of contextuality that is intensively examined outside of physics [6,7,8,9,10]; see [11] for a detailed review and fundamental discussion on context-sensitivity versus contextuality. Before moving to the main topic of the present article, namely experiments on the contextuality of cognition, we would like to present a short introduction to quantum-like modeling and its value for cognitive science.

During the last 15–20 years, the methods of quantum theory started to be applied outside of physics, for example, in cognitive science, psychology, decision making, economics and finance, social and political science, and AI; see, for example, the monographs by Khrennikov [7], Busemeyer and Bruza [12], Haven and Khrennikov [13], Asano et al. [14], Bagarello [15], and a representative collection of articles in Plotnitsky and Haven [10]. Such applications are known as *quantum-like* (or quantum-inspired) models. This area of research should be distanced from the applications of real quantum physics to cognition and consciousness, in the spirit of the works of Hameroff [16] and Penrose [17]. Quantum-like models are applicable to macroscopic systems, e.g., humans and animals. Quantum-like systems process information by following the laws of quantum probability and information and violating the classical laws. But this kind of processing need not (although it might be) reduced to quantum physical processes in the brain, say in microtubules.

The main distinguishing feature of quantum-like information processing by the brain is operating with superpositions of a few values of cognitive entities, i.e., processing unresolved uncertainties [18,19]. Information flowing without value determination saves a lot of computational resources. The brain uses the quantum-like regime for “thinking fast”, e.g., taking decisions quickly; the classical regime is employed for “thinking slow” Kahneman [20]. The latter is based on employing classical Boolean logic and involves operation in a “big Boolean algebra” containing all events related to a cognitive task under processing. By “thinking fast”, the brain does not try to unify all available information in a Boolean algebra; it operates in a bunch of Boolean algebras unified by the structure of an orthomodular lattice, also known as quantum logic [21]. By operating with unresolved uncertainties and consequently violating the laws of Boolean logic, the brain can generate important cognitive effects, for example, the order, conjunction, disjunction, and response replicability effects, and violate the Bayes rule for conditional probability and, hence, Bayesian probability inference [21,22]; see also the aforementioned books and, e.g., Refs. [23,24] for reviews.

One of the main distinguishing features of the quantum theory is its *contextuality*. This is a complex notion, and it is characterized by a variety of exhibitions and formalizations [25]. It is natural to borrow some quantum methods, both theoretical and experimental, for the analysis of cognitive contextuality. The Bell-type inequalities [26] are at the heart of quantum foundations (with the Nobel Prize assigned to Aspect, Clauser, and Zeilinger in 2022 for experimental verification of their violation). In physics, their violations are typically associated with *nonlocality*, the action at a distance. However, the Bell inequalities can also be interpreted as tests of contextuality, as was originally pointed by Bell [26]. Such quantum-like (inspired) tests can be performed for decision making by humans [8,27,28,29,30]. The violation of the Bell inequalities shows the context dependence of human decisions and judgments and, hence, the violation of the laws of classical logic. The degree of the violation of these inequalities quantifies contextuality; see also De Baros et al. [31] on the interrelation of contextuality and indistinguishability.

The Leggett–Garg (LG) inequality is the temporal version of the Bell inequalities testing the conditions of macroscopic realism and noninvasive measurability [32]. The LG inequality can be interpreted as a *contextual probabilistic inequality* [14], in which one combines the data collected in experiments performed for three different contexts. In the original version [32] of the inequality, these contexts have a temporal nature and are given by three pairs of instances; (*t*1, *t*2), (*t*2, *t*3), and (*t*3, *t*4), where *t*1 < *t*2 < *t*3. In this article, we follow [14] in using the LG inequality to check the contextuality of statistical data collected in experimental studies on the bistable perception of cup-like objects to distinguish contextual and noncontextual realisms and, more generally, contextual and noncontextual probabilistic representations. We performed two kinds of experiments, Experiment 1 for physically manipulated contexts and Experiment 2 for social contexts. Both show violation of the LG inequality, and the degree of its violation quantifies contextuality.

Another important output of our experimental study is the coupling of cognitive contextuality with *hysteresis* (see Section 5 on cognitive hysteresis). The hysteresis effect is important for clarifying the interrelation of memory, processing uncertainties, value indetermination, and, at least indirectly, it is coupled with the border effect and non-commutative structure of cognitive entities. The first experiment showing the coupling of quantum-like contextuality with hysteresis was performed by Asano et al. [14]. Our present experiments also demonstrated coupling of contextuality and hysteresis, but for Experiment 2, the situation is not as clear as for Experiment 1, and further studies must be conducted.

We note that, although the LG test of contextuality employed in this article is inspired by quantum theory, this theory by itself is not employed. We also note that pragmatically, the quantum tests are useful as well-defined procedures for quantifying the contextuality level.

## 2. Contextuality and Cyclic Systems with Different Ranks

To explore contextuality, one needs to construct a system that consists of different contexts in which different variables can be measured. Cyclic systems are such systems that are commonly used in the literature [33,34]. A cyclic system is a system in which each observable is measured in precisely two different contexts, and each context includes precisely two variables being measured. The number of contexts contained in the system is the rank of the system, which is the same as the number of properties measured in the system. The cyclic systems that have been used to test contextuality normally include rank-5 [35,36], rank-4 [37,38,39,40], rank-3 [14,32,41,42,43], and rank-2 [44,45,46] systems.

In this article, all variables are binary, with possible values of +1 or −1. We will use $R_{x}^{c}$ to denote the random variable *x* being measured in context *c*. Given two jointly distributed variables $R_{x}^{c}$ and $R_{y}^{c}$ being measured in context *c*, the correlation between the two variables is defined as follows:

(1) $$C_{xy}^{c}=\langle R_{x}^{c}R_{y}^{c}\rangle =P\left(R_{x}^{c}=+1,R_{y}^{c}=+1\right)+P\left(R_{x}^{c}=-1,R_{y}^{c}=-1\right)-P\left(R_{x}^{c}=+1,R_{y}^{c}=-1\right)-P\left(R_{x}^{c}=-1,R_{y}^{c}=+1\right)\;=2\left[P\left(R_{x}^{c}=+1,R_{y}^{c}=+1\right)+P\left(R_{x}^{c}=-1,R_{y}^{c}=-1\right)\right]-1$$

Given this definition, we can conclude that the correlation between two random variables is bounded by [−1, 1]. Consider three jointly distributed variables $R_{x}^{c}$, $R_{y}^{c}$, and $R_{z}^{c}$; the marginal consistency among the three variables implies the following equations:

(2) $$P\left(R_{x}^{c}=x_{c},R_{y}^{c}=y_{c}\right)=\underset{R_{z}^{c}}{\sum }P\left(R_{x}^{c}=x_{c},R_{y}^{c}=y_{c},R_{z}^{c}=z_{c}\right),\;\mathrm{for}\;x_{c},y_{c},z_{c}\in \left\{\pm 1\right\}\;P\left(R_{x}^{c}=x_{c}\right)=\underset{R_{y}^{c}}{\sum }P\left(R_{x}^{c}=x_{c},R_{y}^{c}=y_{c}\right)=\underset{R_{z}^{c}}{\sum }P\left(R_{x}^{c}=x_{c},R_{z}^{c}=z_{c}\right),\;\mathrm{for}\;x_{c},y_{c},z_{c}\in \left\{\pm 1\right\}\;1=\underset{R_{x}^{c}}{\sum }P\left(R_{x}^{c}=x_{c}\right),\;\mathrm{for}\;x_{c}\in \left\{\pm 1\right\}$$

Given Equations (1) and (2), we can derive the Leggett–Garg (LG) inequality as follows (the detailed derivation of the inequality is described in Appendix A):

(3) $$K=C_{xy}^{c}+C_{yz}^{c}-C_{xz}^{c}=1-4\left\{P\left(R_{x}^{c}=1,R_{y}^{c}=-1,R_{z}^{c}=1\right)+P\left(R_{x}^{c}=-1,R_{y}^{c}=1,R_{z}^{c}=-1\right)\right\}\leq 1$$

To explore contextuality, we created a rank-3 cyclic system. Our system has three contexts $c_{1}$, $c_{2}$, and $c_{3}$, and three random variables $R_{x}$, $R_{y}$, and $R_{z}$. Each variable is measured in two contexts and each context includes two variables. This design results in six context-variable combinations: $R_{x}^{c_{1}}$, $R_{y}^{c_{1}}$, $R_{y}^{c_{2}}$, $R_{z}^{c_{2}}$, $R_{z}^{c_{3}}$, and $R_{x}^{c_{3}}$. The K-values used to test the Leggett–Garg (LG) inequality can then be defined as:

(4) $$K_{xyz}^{c_{1}c_{2}c_{3}}=C_{xy}^{c_{1}}+C_{yz}^{c_{2}}-C_{xz}^{c_{3}}=\langle R_{x}^{c_{1}}R_{y}^{c_{1}}\rangle +\langle R_{y}^{c_{2}}R_{z}^{c_{2}}\rangle +\langle R_{x}^{c_{3}}R_{z}^{c_{3}}\rangle$$

Given our experimental design, if contextuality does not exist, i.e., the distribution of each variable does not depend on the context in which the variable is measured, then the value of (4) should also be less than or equal to one. However, if contextuality exists among the contexts, the value of (4) could be larger than 1 or smaller than −1. But because the K-values in (4) are calculated from three correlation coefficients, and each correlation coefficient is bounded by [−1, 1], then the K-values in (4) are also bounded by [−3, 3].

## 3. General Method

### 3.1. Test Stimuli

In the original experiment [14], participants were presented with a list of Schöder stairs and were asked to determine which side was the “front” side of the stair. Critically, the “tilt angle” of the stairs was changed between trials. In their study, the meaning of the word “front” used in the instruction was largely unambiguous. The bistable pattern observed in participants’ responses mainly originated from participants’ ambiguous perception of the stair being presented, not from the meaning of the word “front”.

**Figure 1:**
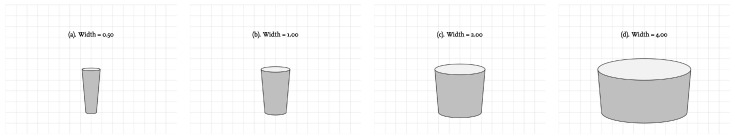
An example of three cup-like objects used in Experiments 1 and 2.

Generally speaking, the relation between a word in human language and its denotation in the physical world is fixed. In some cases, however, objects denoted by certain words, i.e., the semantic space of certain words in human language, could be ambiguous. For example, the object in Figure 1 (especially 1b) could be a cup, a bowl, a mug, or a vase. In this study, we want to utilize the ambiguity of these denotations to explore whether cognition of the human mind violates the Leggett–Garg inequality.

Whether objects like the ones in Figure 1 are regarded as cups or not is affected by the physical properties of the test images, such as the object’s height, the object’s bottom width, and the object’s top width. To simplify things, we fixed the images’ height and the ratio of top width to bottom width and changed their bottom width only. Suppose its height is two units, and the ratio of top width to bottom width is about 1.2; we change its bottom width from 0.50 to 4.00 with a step of 0.02. This results in a series of 176 images, like Figure 1. We have uploaded all images into the “TestImages” folder of the OSF project https://osf.io/rf8s3/ (accessed on 8 June 2024). The images are named as follows: “Cup_W1.00_H2.00_D05.png”, which means its width is 1.00 units, height is 2.00 units, and the ratio of top width to bottom width is “1 + 2cos(05)”, i.e., the tilt angle between the bottom side and left/right side is about 5 degrees. We have uploaded a CSV file called “TestStimuli.csv” to the OSF project to store the relevant information. The CSV file has 177 rows corresponding to the 176 test images, plus a title row. The CSV file has four columns, telling us its bottom width, height, degree, and the name of the test image in that row.

### 3.2. Physical and Social Contexts

The categorization of the objects in Figure 1 is not only affected by the physical properties of the images but also by the context within which the objects are judged. The context includes both the physical context, such as the order of the representation if a series of objects were presented to the same participant (similar to [14]), and the social context under which the judgments are imagined to be made [47]. For example, the same object may be judged differently if described as a coffee container, a food container, or a flower container [48,49,50].

To explore whether participants’ responses violate the Leggett–Garg inequality, three levels of the specific context need to be generated. The current study reports two experiments: In Experiment 1, three levels of physical contexts were manipulated; in Experiment 2, three levels of social context were generated.

In Experiment 1, the social context was kept “neutral”: Participants were simply asked to judge whether the object was a “cup” or not. The following three levels of physical context (three ways to present the stimuli) were manipulated: *decrease* (the fixed order in which the width of the objects decreases from the largest to the smallest one step by one step), *increase* (the fixed order in which the width of the objects increases from the smallest to the largest one step by one step), and *random* (objects are presented in a pseudo-random order).

In Experiment 2, the physical context was kept fixed: Participants were presented with the test stimuli in a pseudo-random order and were asked to imagine that they were in the given social context and to judge whether the object was a cup or not. Three social context levels—*coffee context*, *food context*, and *flower context*—were created in Experiment 2. (1) In the *coffee context*, participants were asked to imagine in each case that they saw someone with the object in his hand, stirring in sugar with a spoon, and drinking coffee from it; (2) In the *food context*, participants were asked to imagine that they came to dinner at someone’s house and saw this object sitting on the dinner table, filled with rice; (3) In the *flower context*, participants were asked to conceive of each of these objects standing on a shelf, each with cut flowers in it.

### 3.3. Experimental Procedure

Both experiments had a between-participant design. Each participant, under a specific level of the given context, was presented with the 176 test images one by one and was asked to judge whether the object was a cup or not by pressing the key “Y” or “N”.

### 3.4. Data Analyses

To analyze the data, we first coded participants’ “Yes” responses as 1 and “No” responses as −1.

Second, if we use $\left[c_{1},c_{2},c_{3}\right]$ to denote the triple experimental context levels (physical context and social context), and use $\left[0.5,0.52,\cdots ,3.98,4.00\right]$ to denote the 176 widths of the images, we then calculate *K*-values using Equation (5):

(5) $$K_{w_{i}w_{j}w_{k}}^{c_{a}c_{b}c_{c}}=C_{w_{i}w_{j}}^{c_{a}}+C_{w_{j}w_{k}}^{c_{b}}-C_{w_{i}w_{k}}^{c_{c}}$$

where $c_{a},c_{b},c_{c}\in \left[c_{1},c_{2},c_{3}\right]$, and $w_{i},w_{j},w_{k}\in \left[0.5,0.52,\cdots ,3.98,4.00\right],\;w_{i}<w_{j}<w_{k}$. The correlation coefficient $C_{w_{x}w_{y}}^{x}$ is calculated using Equation (1). As the three contexts in (5) can be the same or different, a total of $C_{3}^{176}\times 3^{3}=24,116,400$ K-values will be obtained for each experiment. The K-values can actually be divided into three groups. The first group consists of $C_{3}^{176}\times 3=2,679,600$ K-values where the three correlation coefficients used to calculate the K-values are from the same context. Henceforth, the K-values in this group should not be larger than 1. If the K-values in this group exceed one, the validity of the current study should be questioned. The K-values in this group could be used to double-check the validity of our experimental design. The second group consists of $C_{3}^{176}\times 3\times 2\times 3=16,077,600$ K-values, where the three correlation coefficients are calculated from two different contexts. The third group consists of $C_{3}^{176}\times P_{3}^{3}=5,359,200$ K-values, where the three correlation coefficients are calculated from three different contexts. The latter two groups are essential to test the violation of the Leggett–Garg (LG) inequality.

Third, to statistically explore the violation of the Leggett–Garg (LG) inequality, we bootstrapped 500,000 K-values. As we explained earlier, for each triple of the three images being used to calculate K-values, we have six random variables: $R_{w_{i}}^{c_{a}}$, $R_{w_{j}}^{c_{a}}$, $R_{w_{j}}^{c_{b}}$, $R_{w_{k}}^{c_{b}}$, $R_{w_{k}}^{c_{c}}$, $R_{w_{i}}^{c_{c}}$. Each variable has two possible values: +1 or −1. To simulate a K-value, we randomly generate 12 numbers with the following constraints: (1) all the numbers are within the range of [0, 1]; (2) The two variables from the same context are jointly distributed, i.e.,

(6) $$1=\underset{R_{x}^{c},R_{y}^{c}}{\sum }P\left(R_{x}^{c}=x_{c},R_{y}^{c}=y_{c}\right),\;\mathrm{for}\;x_{c},y_{c}\in \left\{\pm 1\right\}$$

We then calculated the 99% quantile of the simulated K-values. If the obtained K-values are larger than the quantile, we can say that the K-values are obtained from a contextual system with a confidence level of 99%. The raw data and the original scripts used to analyze the data and do the plotting were all uploaded to the OSF project.

## 4. Experiment 1

### 4.1. Participants

Ninety-seven native Mandarin speakers from the BLCU were recruited and were paid 15 Chinese Yuan to participate in experiment 1. They were undergraduate or postgraduate students of different majors (age range: 18–25). Informed consent was obtained from each participant, and they were debriefed about the aims of the study after completing the experiment. Thirty-one participants took part in the *decrease* level and were all included in the analyses. Thirty-four participants took part in the *increase* level. Among the 34 participants, one did not finish all the trials, and another two always gave the same answer. We excluded the three participants from the final analyses, resulting in 31 valid participants in the *increase* level. Thirty-two participants were recruited in the *random* level, and one was excluded from the final analyses because she always gave the same answer, resulting in 31 valid participants in the *random* context. We have 31 valid participants in each context level and 93 valid participants in total. We obtained 5456 = 31 × 176 judgments in each context, and 16,386 = 5356 × 3 judgments in total. The raw data and the original scripts used to analyze the data and do the plotting were all uploaded to the OSF project.

### 4.2. Results

**Figure 2:**
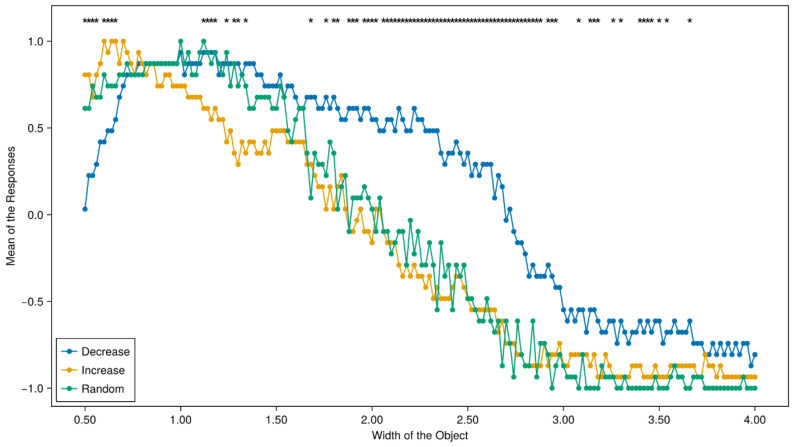
Descriptive results of experiment 1. The *x*-axis is the width of the objects being presented; *the y*-axis is the average responses to the given object. The images with a star placed on top mean that responses to these images are significantly different between the three text contexts (*p* < 0.05).

The effects of physical context (i.e., the presentation order) on participants’ judgments are briefly summarized in Figure 2, where the *x*-axis is the object’s width, and the *y*-axis is the average proportion of “Yes” responses. To test the hysteresis effects, we then conducted a series of 176 statistical tests to see whether participants’ responses to a specific image are affected by the way they are presented. The stars on top of Figure 2 signify that participants’ responses have a significant difference for that specific image (*p* < 0.05). As shown in Figure 2, we found that the perception of the same object as a cup or not a cup was heavily affected by the order in which the objects were presented to the participants.

**Figure 3:**
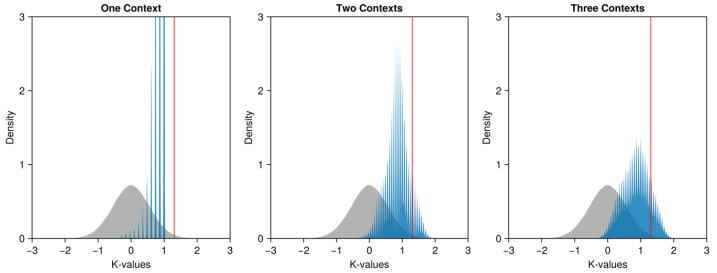
Densities of the K-values obtained in experiment 1. In each panel, the 500_000 simulated K-values are shown as the gray density; the boundary of the 99% quantile is shown as the vertical red line; and the obtained K-values are shown as the blue density. The correlation coefficients are from the same context in the left panel, are from two different contexts in the middle panel, and are from three different contexts in the right panel. The kernel density was calculated from 200 points, the kernel is a normal distribution, and the bandwidth is determined via Silverman’s rule. They are calculated and plotted via the “density” function of the “*CairoMakie.jl*” [51] package under the *Julia* programming language [52].

To explore the violation of the Leggett–Garg (LG) inequality, we plot the obtained K-values in blue against the simulated K-values in gray (Figure 3), with the vertical red line indicating the boundary of the 99% confidence interval. As we can see in the left panel, when the correlation coefficients are from the same context, no observed K-value exceeds the boundary. Actually, among the 2,679,600 K-values, no value exceeds 1. This suggests that the observed K-values between different contexts (Figure 3, middle and right panels) could not solely result from the effects of the specific context, i.e., how the stimuli are presented. To be specific, when the coefficients are from two different contexts (Figure 3, middle panel), 29% (4,717,701 of 16,077,600) of the K-values exceed 1, violating the Leggett–Garg (LG) inequality. Furthermore, 11% (1,807,526 of 16,077,600) of the K-values exceed the boundary of the 99% confidence interval. When the three correlations are obtained from three different contexts (Figure 3, right panel), 36% (1,903,756 of 5,359,200) of the K-values exceed 1, violating the Leggett–Garg (LG) inequality. Furthermore, 16% (859,314 of 5,359,200) of the K-values exceed the boundary of the 99% confidence interval.

## 5. Experiment 2

### 5.1. Participants

Ninety Mandarin-speakers who did not participate experiment 1 were recruited from the BLCU and were paid 15 Chinese Yuan to participate in experiment 2. They were undergraduate or postgraduate students of different majors (age range: 18–25). Informed consent was obtained from each participant, and they were debriefed about the aims of the study after completing the experiment. The ninety participants, thirty participants in each social context level, i.e., *coffee context*, *food context*, and *flower context* are included in the final analyses. We obtained 5280 = 31 × 176 judgments in each context, and 15,840 = 5356 × 3 judgments in total.

### 5.2. Results

**Figure 4:**
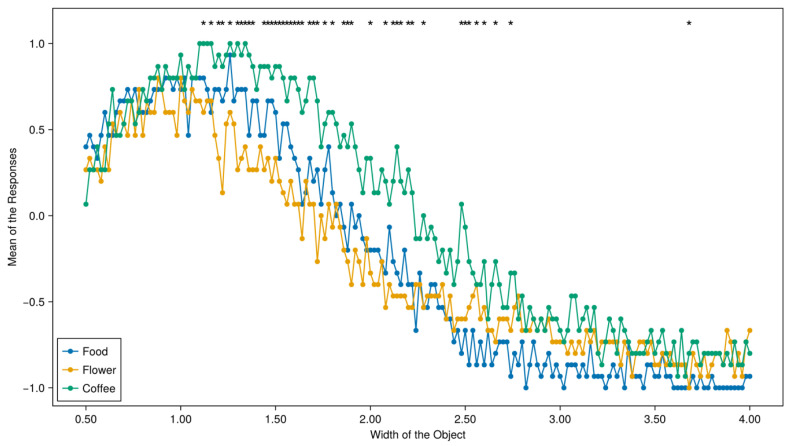
Descriptive results of experiment 2. The *x*-axis is the width of the objects being presented; the *y*-axis is the average responses to the given object. The images with a star placed on top mean that responses to these images are significantly different between the three text contexts (*p* < 0.05).

The raw data and the original scripts used to analyze the data and do the plotting were all uploaded to the OSF project. The effects of social context (i.e., *coffee context*, *food context*, or *flower context*) on participants’ judgments are briefly summarized in Figure 4, where the *x*-axis is the object’s width, and the *y*-axis is the average proportion of “Yes” responses. To test the hysteresis effects, we also conducted a series of 176 statistical tests to see whether participants’ responses to a specific image are affected by the way they are presented. The stars on top of Figure 4 signify that participants’ responses have a significant difference for that specific image (*p* < 0.05). As shown in Figure 4, we found that the perception of the same object as a cup or not a cup was heavily affected by the order in which the objects were presented to the participants. The prevalence of the difference, however, is smaller than that observed in Experiment 1 (Figure 2). This is probably because a significant difference for the “coffee context” is a shift towards higher averaged values of Yes answers for the larger interval of widths. It is understandable because you can drink coffee in cups of many different shapes, unlike eating rice or keeping cut flowers.

**Figure 5:**
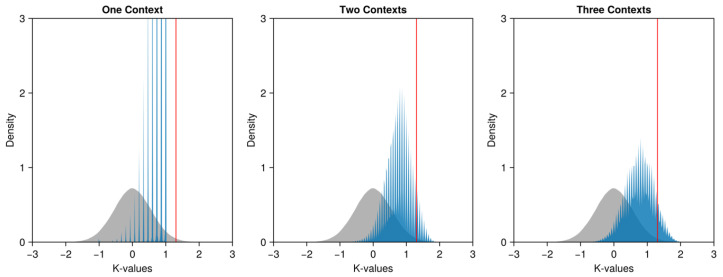
Densities of the K-values obtained in experiment 2. In each panel, the 500_000 simulated K-values are shown as the gray density; the boundary of the 99% quantile is shown as the vertical red line; and the obtained K-values are shown as the blue density. The correlation coefficients are from the same context in the left panel, are from two different contexts in the middle panel, and are from three different contexts in the right panel.

To explore the violation of the Leggett–Garg (LG) inequality, we also plot (Figure 5) the obtained K-values in blue against the simulated K-values in gray, using the vertical red line to indicate the boundary of the 99% confidence interval. We also divide the K-values into three groups. In the left panel, when the correlation coefficients are from the same context, no observed K-value exceeds the boundary. Actually, among the 2,679,600 K-values, no value exceeds 1. When the coefficients are from two different contexts (Figure 5, middle panel), 23% (3,697,450 of 16,077,600) of the K-values exceed 1, violating the Leggett–Garg (LG) inequality. Furthermore, 7% (1,197,509 of 16,077,600) of the K-values exceed the boundary of the 99% confidence interval. When the three correlations are obtained from three different contexts (Figure 5, right panel), 27% (1,473,443 of 5,359,200) of the K-values exceed 1, violating the Leggett–Garg (LG) inequality. Furthermore, 10% (5,359,200 of 5,359,200) of the K-values exceed the boundary of the 99% confidence interval.

## 6. Discussion on Connection with Cognitive Hysteresis

The results reported in our study are related to but are not exactly the same as the hysteresis effects. A system is said to exhibit hysteresis when it responds differently to identical inputs depending on the direction in which the system is being driven [53]. Stimuli used in psychology can normally be ordered via certain dimensions. The stimuli are then presented either in an increasing order or in a decreasing order along that dimension. If the same stimuli are perceived differently when they are presented in different orders, hysteresis effects occur. Hysteresis effects have been found in different perceptions, such as distance perception [54], time perception [55], number perception [56], direction perception [57,58,59,60,61,62], tone perception [63,64,65], emotion perception [61,66,67], object perception [63,64], letter perception [68,69], and language production [70].

In the first experiment of our study, the physical context, i.e., the order along the width of the objects, was manipulated among participants. The experiment found that whether the same object is judged as a cup or not is heavily affected by the order the objects being presented to the participants. The results observed in experiment 1 essentially reveal a kind of hysteresis effect. In the second experiment of our study, the imaged social context was manipulated among participants, with the presenting order being pseudo-randomized. The hysteresis-like effect observed in experiment 2 cannot be directly described as a kind of hysteresis effect. Consequently, the violation of the Leggett–Garg inequality observed in our study cannot be directly ascribed to the hysteresis effect.

## 7. Concluding Remarks

Our experiments confirm the contextuality of human decision making. We employed the test of contextuality inspired by quantum theory, a quantum-like test, seeded in the LG inequality. In quantum physics, the LG inequality is a Bell-like inequality, namely, one of the temporal Bell inequalities. In [14], this temporal inequality was reformulated in contextual terms, and such a contextual version of the LG inequality can be used in the study of human behavior. We performed LG inequality testing for the following two classes of contexts:

**Experiment 1:** Physical contexts are created via manipulation by physical parameters of images.

**Experiment 2:** Social contexts are created via manipulation of the social environment of decision makers.

In both cases, collected statistical data show the contextuality effect that is quantified by the degree of the violation of the contextual LG inequality. Experiment 1 has similarities with the experiment performed in [14]. Experiment 2 is a new step towards testing social contextualization of human decision making with quantum-like tests.

We also studied the cognitive hysteresis effect. For experiment 1, similarly to [14], our data show the hysteresis effect; as was pointed out, the interpretation of our data for experiment 2 is not straightforward, and new experimental studies are needed. We hope that this paper will attract the attention of experts in cognition, psychology, and decision making to the use of quantum-like tests, in the form of various Bell-type inequalities, for the analysis of the dependence of human decisions on socio-physical contexts. Our experiments confirm the contextuality of human decision making. It is clearly visible in our Figure 2 and Figure 4, where sample means for 176 measured random variables are displayed and compared.

Quantum contextuality is usually assessed using various noncontextuality inequalities, e.g., Bell–CHSH or Leggett–Garg. Inspired by these tests, [14] reformulated the temporal LG inequality in contextual terms, and such a contextual version of the LG inequality can be used in the study of human behavior. To quantify cognitive contextuality in our experiments, we also studied the degree of the violation of Asano–LG inequalities. However, caution is needed in drawing far-reaching conclusions from the violation of Bell-type inequalities in physics and in cognitive science. The violation of these inequalities only allows the rejection a statistical hypothesis that all the experimental data from experiments performed in different contexts are described by a unique set of contextual jointly distributed random variables. In physics, it does not prove the nonlocality of nature, and in social and cognitive sciences, it does not give a lot of information about human cognition or decision making. For a detailed explanation, see [71,72,73,74,75]. In Bell tests performed in physics, we have data from 3 or 4 experiments outputting only pairs of outcomes of 1 or −1. In our experiments studying the bi-stable perception of objects, we know the full empirical probability distributions of 176 random variables measured in different contexts. By studying and comparing these distributions using standard nonparametric tests, we can obtain much more detailed and reliable information about contextuality in human cognition than we can obtain from Bell-type and Asano–LG tests.

## Data Availability

Data is contained within the article.

## Appendix A. Proof of Equation (3)

(A1)
$$\begin{array}{c} K\\ =C_{xy}^{c}+C_{yz}^{c}-C_{xz}^{c}\\ =\left\{2\left[P\left(R_{x}^{c}=1,R_{y}^{c}=1\right)+P\left(R_{x}^{c}=-1,R_{y}^{c}=-1\right)\right]-1\right\}+\\ \;\;\;\;\left\{2\left[P\left(R_{y}^{c}=1,R_{z}^{c}=1\right)+P\left(R_{y}^{c}=-1,R_{z}^{c}=-1\right)\right]-1\right\}-\\ \;\;\;\;\left\{2\left[P\left(R_{x}^{c}=1,R_{z}^{c}=1\right)+P\left(R_{x}^{c}=-1,R_{z}^{c}=-1\right)\right]-1\right\}\\ =\{2[P\left(R_{x}^{c}=1,R_{y}^{c}=1,R_{z}^{c}=1\right)+P\left(R_{x}^{c}=1,R_{y}^{c}=1,R_{z}^{c}=-1\right)+\\ \;\;\;\;\;P\left(R_{x}^{c}=-1,R_{y}^{c}=-1,R_{z}^{c}=1\right)+P\left(R_{x}^{c}=-1,R_{y}^{c}=-1,R_{z}^{c}=-1\right)]-1\}+\\ \;\;\;\;\;\{2[P\left(R_{y}^{c}=1,R_{z}^{c}=1,R_{x}^{c}=1\right)+P\left(R_{y}^{c}=1,R_{z}^{c}=1,R_{x}^{c}=-1\right)+\\ \;\;\;\;\;\;P\left(R_{y}^{c}=-1,R_{z}^{c}=-1,R_{x}^{c}=1\right)+P\left(R_{y}^{c}=-1,R_{z}^{c}=-1,R_{x}^{c}=-1\right)]-1\}-\\ \;\;\;\;\;\;\{2[P\left(R_{x}^{c}=1,R_{z}^{c}=1,R_{y}^{c}=1\right)+P\left(R_{x}^{c}=1,R_{z}^{c}=1,R_{y}^{c}=-1\right)+\\ \;\;\;\;\;\;P\left(R_{x}^{c}=-1,R_{z}^{c}=-1,R_{y}^{c}=1\right)+P\left(R_{x}^{c}=-1,R_{z}^{c}=-1,R_{y}^{c}=-1\right)]-1\}\\ =\{2[P\left(R_{x}^{c}=1,R_{y}^{c}=1,R_{z}^{c}=1\right)+P\left(R_{x}^{c}=1,R_{y}^{c}=1,R_{z}^{c}=-1\right)+\\ \;\;\;\;\;\;P\left(R_{x}^{c}=-1,R_{y}^{c}=-1,R_{z}^{c}=1\right)+P\left(R_{x}^{c}=-1,R_{y}^{c}=-1,R_{z}^{c}=-1\right)]-1\}+\\ \;\;\;\;\;\{2[P\left(R_{x}^{c}=1,R_{y}^{c}=1,R_{z}^{c}=1\right)+P\left(R_{x}^{c}=-1,R_{y}^{c}=1,R_{z}^{c}=1\right)+\\ \;\;\;\;\;P\left(R_{x}^{c}=1,R_{y}^{c}=-1,R_{z}^{c}=-1\right)+P\left(R_{x}^{c}=-1,R_{y}^{c}=-1,R_{z}^{c}=-1\right)]-1\}-\\ \;\;\;\;\{2[P\left(R_{x}^{c}=1,R_{y}^{c}=1,R_{z}^{c}=1\right)+P\left(R_{x}^{c}=1,R_{y}^{c}=-1,R_{z}^{c}=1\right)+\\ \;\;\;\;P\left(R_{x}^{c}=-1,R_{y}^{c}=1,R_{z}^{c}=-1\right)+P\left(R_{x}^{c}=-1,R_{y}^{c}=-1,R_{z}^{c}=-1\right)]-1\}\\ =\{2P\left(R_{x}^{c}=1,R_{y}^{c}=1,R_{z}^{c}=1\right)+2P\left(R_{x}^{c}=1,R_{y}^{c}=1,R_{z}^{c}=-1\right)+\\ \;\;\;\;2P\left(R_{x}^{c}=-1,R_{y}^{c}=-1,R_{z}^{c}=1\right)+2P\left(R_{x}^{c}=-1,R_{y}^{c}=-1,R_{z}^{c}=-1\right)-1\}+\\ \;\;\;\;\{2P\left(R_{x}^{c}=1,R_{y}^{c}=1,R_{z}^{c}=1\right)+2P\left(R_{x}^{c}=-1,R_{y}^{c}=1,R_{z}^{c}=1\right)+\\ \;\;\;\;2P\left(R_{x}^{c}=1,R_{y}^{c}=-1,R_{z}^{c}=-1\right)+2P\left(R_{x}^{c}=-1,R_{y}^{c}=-1,R_{z}^{c}=-1\right)-1\}-\\ \;\;\;\;\{2P\left(R_{x}^{c}=1,R_{y}^{c}=1,R_{z}^{c}=1\right)+2P\left(R_{x}^{c}=1,R_{y}^{c}=-1,R_{z}^{c}=1\right)+\\ \;\;\;\;\;2P\left(R_{x}^{c}=-1,R_{y}^{c}=1,R_{z}^{c}=-1\right)+2P\left(R_{x}^{c}=-1,R_{y}^{c}=-1,R_{z}^{c}=-1\right)-1\}\\ =\{2P\left(R_{x}^{c}=1,R_{y}^{c}=1,R_{z}^{c}=1\right)+2P\left(R_{x}^{c}=1,R_{y}^{c}=1,R_{z}^{c}=-1\right)+\\ \;\;\;\;\;2P\left(R_{x}^{c}=-1,R_{y}^{c}=-1,R_{z}^{c}=1\right)+2P\left(R_{x}^{c}=-1,R_{y}^{c}=-1,R_{z}^{c}=-1\right)-1\}+\\ \;\;\;\;\;\left\{2P\left(R_{x}^{c}=-1,R_{y}^{c}=1,R_{z}^{c}=1\right)+2P\left(R_{x}^{c}=1,R_{y}^{c}=-1,R_{z}^{c}=-1\right)\right\}-\\ \;\;\;\;\;\left\{2P\left(R_{x}^{c}=1,R_{y}^{c}=-1,R_{z}^{c}=1\right)+2P\left(R_{x}^{c}=-1,R_{y}^{c}=1,R_{z}^{c}=-1\right)\right\}\\ =2P\left(R_{x}^{c}=1,R_{y}^{c}=1,R_{z}^{c}=1\right)+2P\left(R_{x}^{c}=1,R_{y}^{c}=1,R_{z}^{c}=-1\right)+\\ \;\;\;\;\;2P\left(R_{x}^{c}=-1,R_{y}^{c}=-1,R_{z}^{c}=1\right)+2P\left(R_{x}^{c}=-1,R_{y}^{c}=-1,R_{z}^{c}=-1\right)+\\ \;\;\;\;\;2P\left(R_{x}^{c}=-1,R_{y}^{c}=1,R_{z}^{c}=1\right)+2P\left(R_{x}^{c}=1,R_{y}^{c}=-1,R_{z}^{c}=-1\right)-\\ \;\;\;\;\;2P\left(R_{x}^{c}=1,R_{y}^{c}=-1,R_{z}^{c}=1\right)-2P\left(R_{x}^{c}=-1,R_{y}^{c}=1,R_{z}^{c}=-1\right)-1\\ =2-4P\left(R_{x}^{c}=1,R_{y}^{c}=-1,R_{z}^{c}=1\right)-4P\left(R_{x}^{c}=-1,R_{y}^{c}=1,R_{z}^{c}=-1\right)-1\\ =1-4\left\{P\left(R_{x}^{c}=1,R_{y}^{c}=-1,R_{z}^{c}=1\right)+P\left(R_{x}^{c}=-1,R_{y}^{c}=1,R_{z}^{c}=-1\right)\right\}\\ \leq 1 \end{array}$$
